# Coexistence of valley polarization and Chern insulating states in MoS_2_ monolayers with n-p codoping

**DOI:** 10.1038/s41598-020-66912-y

**Published:** 2020-06-17

**Authors:** Xinyuan Wei, Jiayong Zhang, Bao Zhao, Zhongqin Yang

**Affiliations:** 1 State Key Laboratory of Surface Physics and Key Laboratory of Computational Physical Sciences (MOE) & Department of Physics, Fudan University, Shanghai, 200433 China; 2 Jiangsu Key Laboratory of Micro and Nano Heat Fluid Flow Technology and Energy Application, School of Mathematics and Physics, Suzhou University of Science and Technology, Suzhou, Jiangsu 215009 China; 3 School of Physics Science and Information Technology, Shandong Key Laboratory of Optical Communication Science and Technology, Liaocheng University, Liaocheng, Shandong 252059 China; 4 Collaborative Innovation Center of Advanced Microstructures, Fudan University, Shanghai, 200433 China

**Keywords:** Topological insulators, Spintronics

## Abstract

The electronic and topological properties of MoS_2_ monolayers with n-p codoping effect are investigated by using first-principles calculations. Two types of the doped Nb atoms play the roles of the p-type and n-type dopants, respectively. The n-p codoping is found inducing a large valley polarization, associated with the strong magnetization induced by the Nb dopants. Interestingly, the system simultaneously owns a perfect Chern insulating band gap opened exactly at the Fermi level. The nontrivial band gap comes from the lifting of the degeneracy of the d_xz_ and d_yz_ orbitals of Nb_2_ atoms after the spin-orbit coupling is considered. Our work inspires exciting prospects to tune the novel properties of materials with n-p codoping effects.

## Introduction

Recently, transition-metal dichalcogenides (TMDs) have been proposed as excellent candidates for electronics, spintronics, and valleytronics materials by manipulating the charge, spin, and valley degrees of freedom in the system^1–4^. For example, the experimental realization of valley polarization could be through optical pumping^5,6^ in MoS_2_ monolayers (MLs) or externally applied magnetic fields^7–9^ in WSe_2_ and MoSe_2_ monolayers. The approach of optical pumping is, however, restricted by the limited carrier lifetimes in dynamical process. And the valley polarization achieved through an external magnetic field is generally quite small. An alternative way to control the valley degree of freedom in TMDs is through magnetic atom doping^10,11^ or the proximate effect from magnetic substrates^12^.

N-p codoping, with both n-type and p-type dopants in one material, has been proved to be an effective strategy to tune the electronic properties^13–15^. Ferromagnetic (FM) order was reported in graphene with Ni-B codoping^13^. And quantum anomalous Hall effect was predicted in graphene^14^ and Sb_2_Te_3_^15^ through n-p codoping. In this work, we explore the electronic structures and valleytronics in the MoS_2_ monolayer with n-p codoping. Very large valley polarization at the MoS_2_ valence bands is obtained, attributed to the imbalance of K and K′ bands aroused by the magnetic Nb dopants. Chern insulating states are also found in the system. The coexistence of valley polarization and Chern insulating effects in the MoS_2_ ML with Nb n-p codoping demonstrates that this kind of system has potential applications in not only valleytronics, but also electronics and spintronics, which will greatly facilitate the device integration in practice.

## Results and discussion

**Figure 1:**
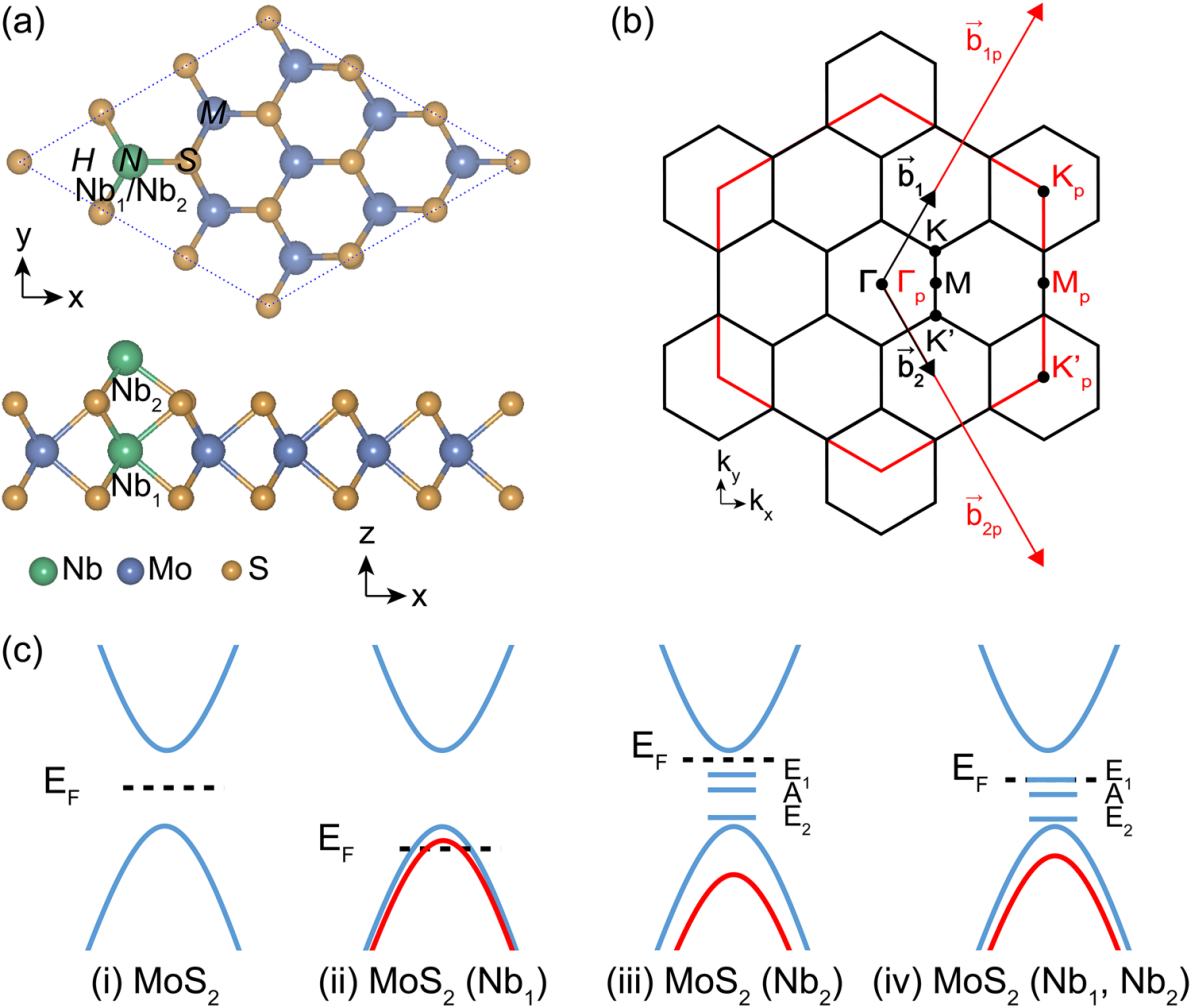
(**a**) Top and side views of the 3 × 3 supercell of the MoS_2_ ML with Nb n-p codopants. The side view is for the *N* configuration. (**b**) Reciprocal momentum space structure, the red and black hexagons/arrows are the reciprocal lattices/vectors for the 1 × 1 and 3 × 3 supercells, respectively. The special k-points for the 1 × 1 supercell are marked with subscript p. (**c**) Evolution of the band structures for the pristine MoS_2_ ML (i) and the Nb_1_ doped (ii), Nb_2_ doped (iii), and Nb_1_-Nb_2_ codoped (iv) MoS_2_ MLs.

For the 3 × 3 supercell MoS_2_ ML with one Mo substituted by Nb atom (Nb_1_), the Nb tends to substitute the Mo atom instead of the S atom for both rich Mo and S cases, consistent with the tendency reported in previous researches^16–20^. The Nb_1_ substituted MoS_2_ ML is also proved to be dynamically stable (without imaginary frequency) through the density functional perturbation theory (DFPT)^21^. Based on this structure, four typical high-symmetry adsorption sites are considered for Nb_2_ atom, with the adsorption energy calculated as: ${E}_{{\rm{a}}}={E}_{{\rm{sample}}}+{E}_{{\rm{adatom}}}-{E}_{{\rm{total}}}$. The *E*_sample_, *E*_adatom_, and *E*_total_ are the total energies of the MoS_2_ ML with Nb_1_ doping, the single Nb atom, the MoS_2_ ML with Nb_1_-Nb_2_ codoping, respectively. As shown in Table 1, the most stable configuration is *N* (see Fig. 1(a)). For comparison, the obtained adsorption energy of the most stable configuration *M* for the pristine MoS_2_ ML with Nb_2_ adsorption is also given in Table 1. As the definition indicates, the adsorption energy reflects the interaction strength between the Nb_2_ atom and the two-dimensional sheet. For the pristine MoS_2_ ML, the adsorption energy is 2.485 eV, while if the sheet is the Nb_1_ doped MoS_2_ ML (Fig. 1(a)), the adsorption energy increases to 3.789 eV. Thus, we infer that the interaction strength of the Nb_2_ adatom in the Nb_1_-Nb_2_ codoping case is enhanced (by 1.3 eV). This behavior can be ascribed to the strong electrostatic attractive interaction between the n-type (Nb_2_) and p-type (Nb_1_) dopants. In the following, we primarily focus on the most stable *N* configuration for the Nb_1_-Nb_2_ codoping case.

**Table 1:** The adsorption energies (E_a_), total and local magnetic moments (M_T_, M_Nb1_, M_Nb2_) in the MoS_2_ MLs with Nb_1_-Nb_2_ codoping or only Nb_2_ doping.

Doping Type	Site	E_a_ (eV)	M_T_ (μ_B_)	M_Nb1_ (μ_B_)	M_Nb2_ (μ_B_)
Nb_1_-Nb_2_ codoping	*H*	3.408	4.00	0.05	2.76
	*M*	3.750	4.00	0.01	2.44
	***N***	**3.789**	**4.00**	**0.05**	**2.49**
	*S*	−0.065	3.80	0.09	2.37
Nb_2_ doping	***M***	**2.485**	**5.00**	***/***	**2.53**

The most stable configurations are marked in bold.

**Figure 2:**
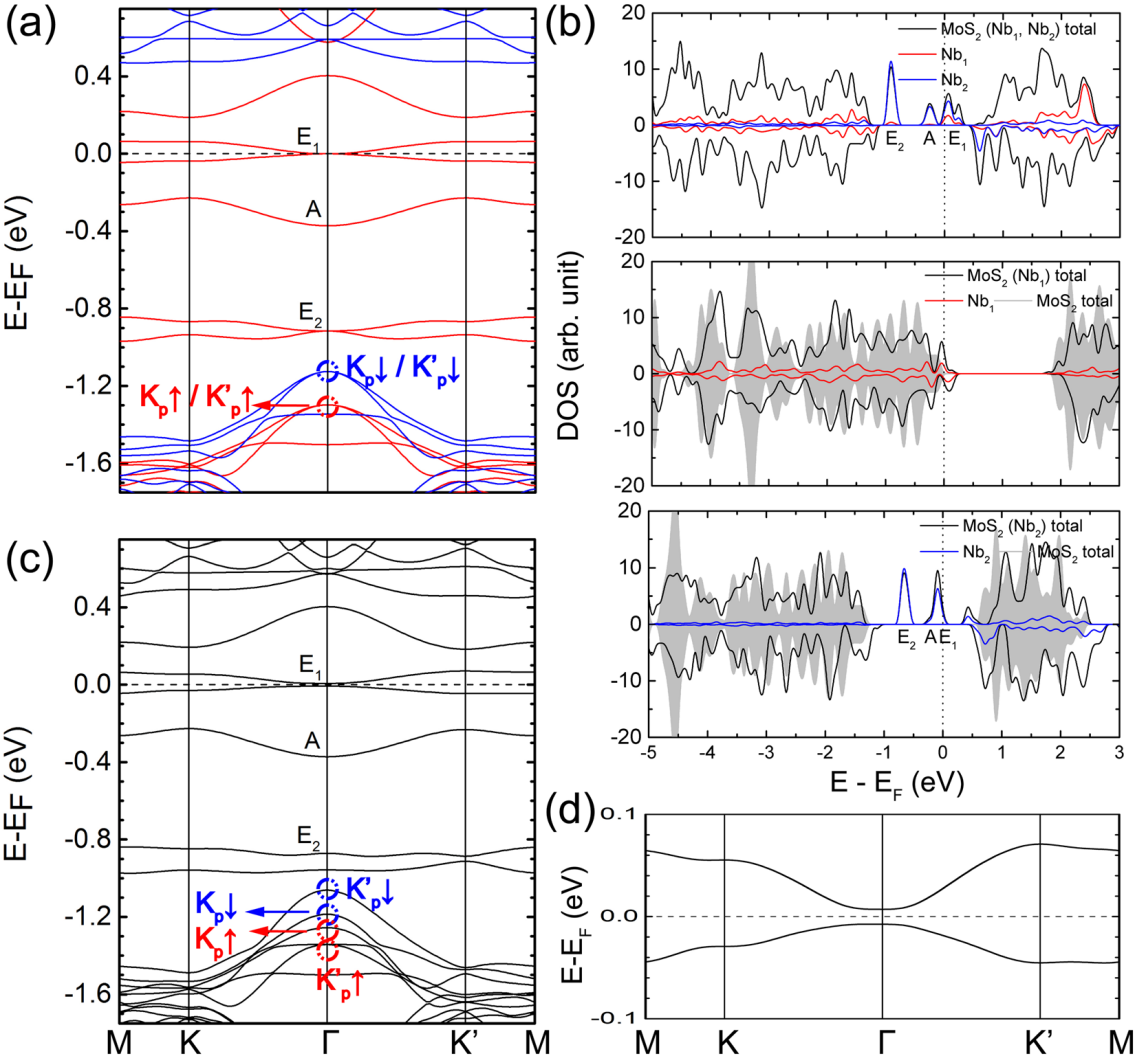
(**a**) Band structure of the Nb_1_-Nb_2_ codoped MoS_2_ ML without SOC. The red and blue curves show the spin-up and spin-down components, respectively. The black curves in the upper, middle, and lower panels of (**b**) are the total DOSs of the Nb_1_-Nb_2_ codoped, Nb_1_ doped, and Nb_2_ doped MoS_2_ MLs, respectively. The red/blue curves are the partial DOSs of the Nb_1_/Nb_2_ atom. The gray areas in the middle and lower panels of (**b**) are the total DOSs of the pristine MoS_2_ ML. The Nb_1_ and Nb_2_ partial DOSs are quadrupled and doubled to obtain a clear view. The positive and negative values correspond to the spin-up and spin-down components, respectively. (**c**,**d**) are the normal and enlarged views of the band structures of the Nb_1_-Nb_2_ codoped MoS_2_ ML with SOC. The small red/blue circles in (**a**,**c**) indicate the spin-up/spin-down component located around Γ, folded from the K_p_ and K′_p_ in the primitive cell.

The band structure and the densities of states (DOSs) for the MoS_2_ ML with Nb_1_-Nb_2_ codoping are given in Fig. 2(a) and the upper panel of Fig. 2(b), respectively. The spin-orbit coupling (SOC) is not yet considered. The Nb_1_ DOSs distribute over a wide range of energy, similar to those of Mo atoms in the pristine MoS_2_^22^, indicating strong bonds formed between Nb_1_ and its neighboring S atoms. The DOSs of the Nb_2_ are, however, mainly located within the band gap of the pristine MoS_2_. To further comprehend the roles of the two types of Nb atoms, the electronic structures of the pristine MoS_2_ ML with solely Nb_1_ or Nb_2_ are also calculated. For the MoS_2_ ML with one Mo atom substituted by one Nb atom in the 3 × 3 supercell, labeled as MoS_2_ (Nb_1_), the Fermi level (E_F_) is now located below the top of the valence bands of the pristine MoS_2_ (middle panel of Fig. 2(b)), implying the p-type acceptor character of Nb_1_ dopant. When one Nb atom is adsorbed on the top of one of the Mo atoms of the pristine MoS_2_ ML, marked as MoS_2_ (Nb_2_), the E_F_ of the system is very close to the bottom of the conduction bands of the pristine MoS_2_ (lower panel of Fig. 2(b)). Thus, Nb_2_ adatom acts as the n-type donor. The *d* orbital of Nb_2_ atom splits into A (d_z2_), E_1_ (d_xz_, d_yz_), and E_2_ (d_xy_, d_x2−y2_) (Fig. 2(a,b)), due to the C_3v_ symmetry owned.

The band evolution from the pristine MoS_2_ ML to the Nb_1_-Nb_2_ codoped MoS_2_ ML is summarized in Fig. 1(c). For the pristine MoS_2_ ML, the E_F_ is within the band gap. For the MoS_2_ (Nb_1_) case, the E_F_ moves downwards to the valence bands of the pristine MoS_2_, while for the MoS_2_ (Nb_2_) case, the E_F_ moves upper to the conduction bands of the pristine MoS_2_, indicating Nb_1_ and Nb_2_ act as p-type and n-type dopants, respectively. For the MoS_2_ (Nb_1_, Nb_2_) case, the compensation effect of the n-type and p-type doping causes the E_F_ located exactly at the E_1_ bands.

Besides the movements of the E_F_ position, the magnetic behaviors of the Nb_1_ and Nb_2_ atoms in the systems are also different from each other. For example, the MoS_2_ (Nb_1_) is weakly spin polarized with a total magnetic moment of 0.73 μB (the middle panel in Fig. 2(b)), while the total magnetic moment is 5.00 μB for the MoS_2_ (Nb_2_). The strong magnetism of the MoS_2_ (Nb_2_) can be comprehended through strong atomic behavior of Nb_2_ atoms, consistent with the case of the *3d* transition metal atom adsorption in MoS_2_ MLs in previous studies^23,24^. For the Nb n-p codoping case, the total magnetic moment of 4.00 μB, instead of 5.00 μB, is obtained for the most stable *N* configuration. The decrease of the magnetism in the n-p codoping case can be ascribed to the p-type Nb_1_ in the n-p codoping case gaining one unpaired electron from Nb_2_. The local magnetic moments of the Nb_1_ and Nb_2_ in the n-p codoped system are about 0.05 μB and 2.49 μB, respectively. Thus, the magnetism in the n-p codoped MoS_2_ ML is primarily induced by the Nb_2_ adatoms. To find whether the above FM structure is the magnetic ground state, the total energies of non-magnetic (NM) and antiferromagnetic (AFM) structures are also calculated. The total energy of the FM ordering per Nb_1_-Nb_2_ pair is found to be lower than that of the NM ordering by 355 meV and AFM ordering by 2 meV. Thus, the FM ordering is the most stable magnetic configuration for the Nb codoped system. The Curie temperature (*T*_*c*_) estimated with the mean-field approximation^25^ is about 15.5 K.

The C_3v_ symmetry owned by the system makes the E_1_ (d_xz_, d_yz_) and E_2_ (d_xy_, d_x2−y2_) bands both be degenerate at the Γ point with quadratic non-Dirac band dispersions before the SOC is included (Fig. 2(a)). Under this case, the system could be called as a spin-gapless semiconductor (SGS)^26–28^ for the bands at the E_F_ are 100% spin polarized. When the SOC is included, the doubly degenerate energy points at the Γ point are lifted and band gaps are opened, as shown in Fig. 2(c,d). Additionally, the degeneracy of the tops of the valence bands of the pristine MoS_2_ (around −1.2 eV in Fig. 2(a)) is also lifted (Fig. 2(c)). E_1_ is exactly located at the E_F_ without the SOC (Fig. 2(a)) and a global band gap of 15.0 meV is opened by the SOC in the system (Fig. 2(d)). We also check the results by using the HSE06 functional^29^. The calculations show that the main feature keeps undistributed with the HSE06 functional, i.e., the E_1_ bands are still located around the E_F_ and a global band gap is also opened. The opened band gap is 36.4 meV, larger than the result obtained from the metaGGA method, due to the stronger exchange-correlation interactions predicted for the Nb d_xz_ and d_yz_ orbitals in the HSE06 functional. The real value of the band gap may be determined by future experimental measurements. Hence, the n-p codoped MoS_2_ ML is an insulator. As shown in Fig. 1(c), if there is no Nb_1_ in the system, the E_1_ bands are totally occupied by electrons. It is, thus, the Nb n-p codoping that gives rise to the E_1_ bands located exactly at the E_F_.

**Figure 3:**
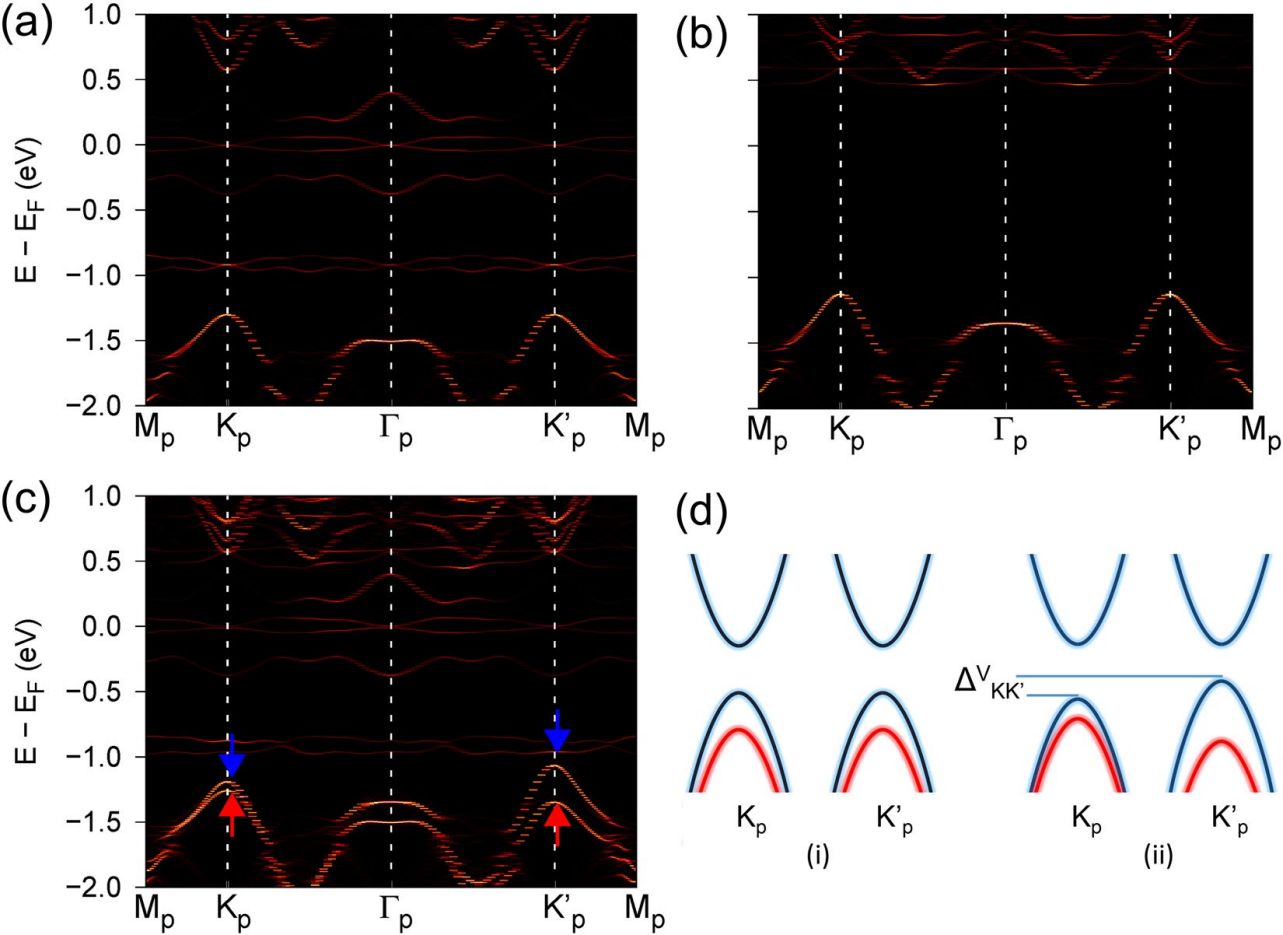
Unfolded band structures for the Nb n-p codoped MoS_2_ ML. (**a**,**b**) are for the spin-up and spin-down bands without SOC, respectively. (**c**) is for the SOC calculation. The small red/blue arrow indicates the spin-up/spin-down bands. (**d**) Schematic diagrams of the bands around the K_p_ and K′_p_ points without (i) and with (ii) SOC in the primitive cell. The red and blue curves represent the bands with different spin components.

Due to the strong magnetism of the Nb_2_ adatoms, legible spin polarization of about 0.2 eV is induced at the tops of the valence bands of MoS_2_ (around −1.2 eV in Fig. 2(a)). This magnetism together with the broken space-inversion symmetry in the system may lead to valley polarization effect^30,31^. However, the use of the 3 × 3 supercell in the calculations causes band folding. To explore the valley polarization of the n-p codoped MoS_2_ ML, the bands shown in Fig. 2(a,c) are unfolded onto the k-points in the Brillouin zone (BZ) of the 1 × 1 primitive cell (Fig. 1(b)) by using a k-projection method^32–34^. Figure 3(a,b) show the unfolded bands for the Nb n-p codoped MoS_2_ ML without SOC. The obvious spin polarization in the valence bands of the MoS_2_ is also outstanding. The bands at K_p_ and K′_p_ for both the spin-up and spin-down components are degenerate, whose sketches are displayed in the (i) case of Fig. 3(d). Thus, no valley polarization appears in the bands.

When the SOC is included, a valley polarization of 125 meV can be observed, defined as $\Delta {V}_{K{K}^{{\rm{{\prime} }}}}={E}_{{K{\rm{{\prime} }}}_{p}}^{V}-{E}_{{K}_{p}}^{V}$. When the HSE06 functional is employed, an almost same value of the valley polarization is obtained, due to the small exchange-correlation interaction for the Mo d orbitals mainly contributing to the valley bands  in the system. Only in the unfolded bands (Fig. 3(c)), the valley polarization can be distinguished. How the tops of the valence bands of the MoS_2_ in Fig. 2(c) correspond to the K_p_ or K′_p_ points in the MoS_2_ primitive cell is also illustrated. Figure 3(d) shows the schematic diagrams of the valley polarization formed in the Nb n-p codoped MoS_2_ ML. Reference^12^ has found that the magnitude of the valley polarization is limited by the smaller spin splitting arising from the SOC or the exchange field. Therefore, the valley polarization obtained (125 meV) should be limited by the smaller SOC strength other than the spin polarization (200 meV). The valley polarization may be enhanced further with the increase of the SOC, by such as substituting the atoms with heavier elements. The obtained valley polarization is actually an intrinsic attribute owned by the system and no magnetic field or optical pumping is necessary to realize the effect. This valley polarization makes the Nb n-p codoped MoS_2_ ML can be called as a ferrovalley material. If the spin orientations of the Nb atoms are reversed, the band features at K and K′ are exchanged, similar to the case in ref. ^35^. Thus, the ferrovalley property still exists in the system, with the same valley polarization strength, but an opposite sign. Since the total energies of the two systems are the same, the two ferrovalley states can be regarded as energy degenerate states.

**Figure 4:**
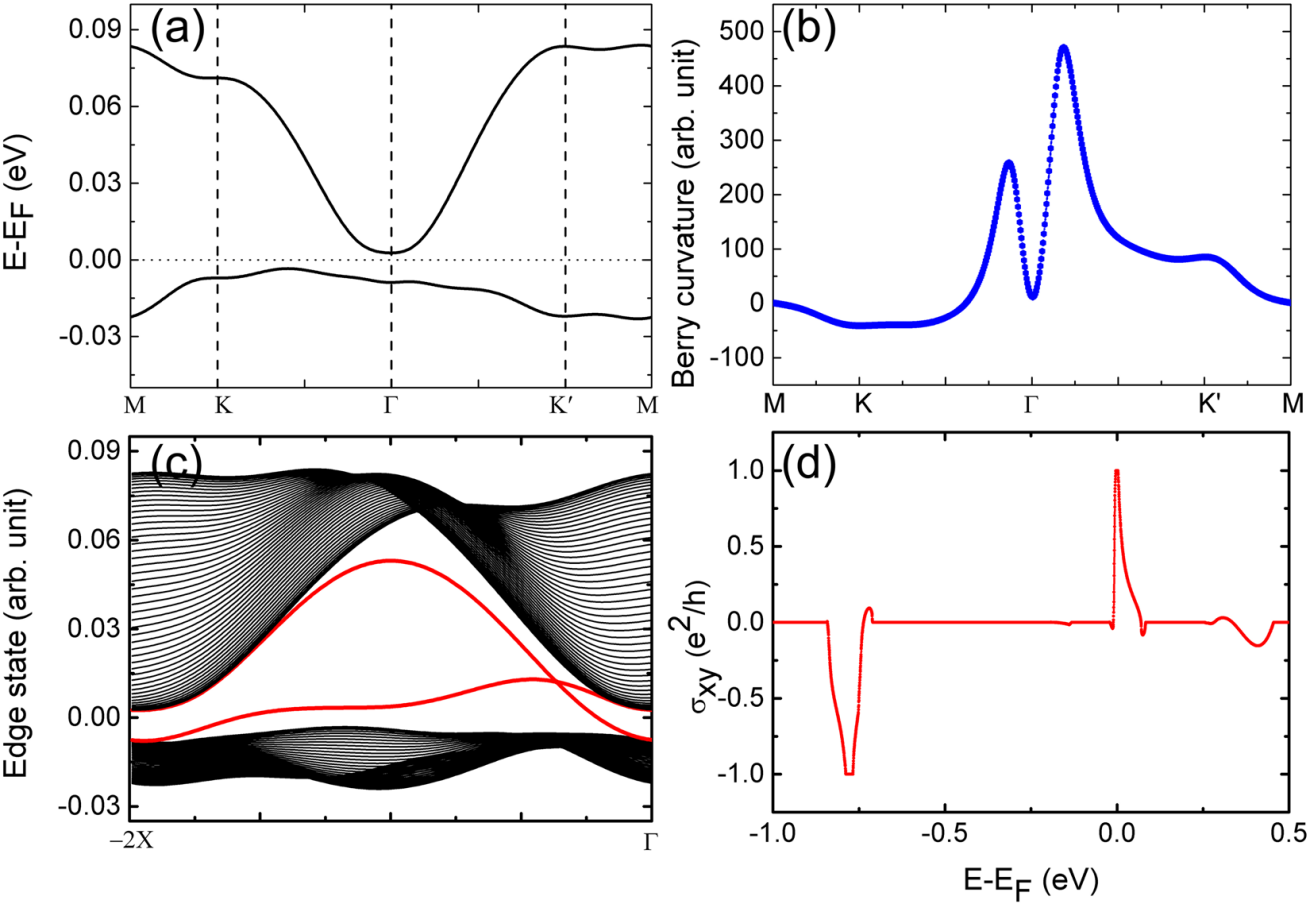
Wannier-function fitted bands around the E_F_ (**a**), Berry curvatures (**b**), edge states (**c**), and Hall conductivity (**d**) for the Nb_1_-Nb_2_ codoped MoS_2_ ML.

We now identify the possible topological behaviors of the band gap opened at the E_F_ (Fig. 2(d)). The bands are fitted by using maximally localized Wannier functions (MLWFs) method^36–38^ (Fig. 4(a)). The Berry curvatures can then be calculated. Two peaks of the Berry curvatures appear around the Γ point (Fig. 4(b)), ascribed to the parabolic band dispersions of E_1_ (Fig. 2(a)) without SOC^39^. The calculated Chern number of 1 gives a direct evidence for the existence of Chern insulating state in the system. Figure 4(c) displays the band structure of a one-dimensional Nb n-p codoped MoS_2_ nanoribbon, calculated by using the tight-binding model constructed from the MLWFs. The two edge states (red curves in Fig. 4(c)) connecting the conduction and valence bands exist inside the SOC induced band gap, representing one chiral dissipationless conducting channel existing on each side of the nanoribbon sample. Besides, the edge states should be 100% spin-polarized and half-metallic since the two-fold E_1_ bands in Fig. 2(a) are spin up^40,41^, availing the applications of the Nb n-p codoped MoS_2_ ML system in spintronic devices. The quantized Hall conductivity of e^2^/h at E_F_ (Fig. 4(d)) matches well with the nontrivial band gap contributed by E_1_ orbitals, while the Hall conductivity of −e^2^/h at −0.8 eV appears due to the E_2_ orbitals. The forming mechanism of these topological states can be ascribed to the SOC-induced band splitting of the degenerate quadratic non-Dirac bands at the Γ point^39–41^. Thus, the large ferrovalley effect and the Chern insulating state achieved in the Nb n-p codoped MoS_2_ monolayer are contributed from the bands at the K (K′) and Γ points in the momentum space, respectively.

The electronic and topological properties of the Nb codoped systems with different Nb doping concentrations are also studied. If the Nb doping concentration is increased, such as one pair of Nb_1_-Nb_2_ doped in one 2 × 2 supercell, the band structure is found being changed much and becomes metallic, ascribed to the strong interaction between the different Nb pairs. If the Nb doping concentration is lowered, for example, one pair of Nb_1_-Nb_2_ doped in one 4 × 4 supercell, the valley polarization and the nontrivial bands can still be clearly observed, although global band gaps may not be opened. Therefore, the Nb doping concentration should be carefully examined to give perfect Chern insulting states and large valley polarization in the MoS_2_ MLs.

In experiments, the n-p codoping has been realized in some materials, such as dilute magnetic semiconductors of ZnMnAlO and ZnCoAlO^42^. Based on these techniques, the n-p codoped MoS_2_ ML may be fabricated by following two steps. First, synthesize the p-type doped MoS_2_ ML with Nb_1_ atoms substituting the Mo atoms via e-beam evaporation plus chemical vapor deposition (CVD), as implemented in ref. ^16^ or chemical vapor transport (CVT) in refs. ^17,18^. Second, deposit some Nb atoms onto the surface of the prepared p-type MoS_2_ ML. Due to the electrostatic attraction between the n-type and p-type dopants, these deposited Nb atoms (Nb_2_) tend to be located on the top of the Nb_1_ atoms, as the obtained adsorption energies indicate. In this way, the Nb n-p codoped system might be achieved in experiments.

## Conclusion

The electronic states of the Nb n-p codoped MoS_2_ monolayer are studied with *ab initio* calculations. Two Nb atoms (Nb_1_ and Nb_2_) serve as p-type and n-type dopants, respectively. Large valley polarization is predicted, caused by the induced magnetism together with the broken space-inversion symmetry and the SOC interaction in the MoS_2_ system. The Nb n-p codoped MoS_2_ monolayer is also a Chern insulator, whose edge can conduct the pure spin-up current without energy dissipation. The coexistence of the large valley polarization and Chern insulting states provides encouraging routes in applying the codoped two-dimensional materials in the fabrication of valleytronic, microelectronic, and spintronic devices.

## Methods

Since experimental studies have shown that the Mo atoms in MoS_2_ thin films can be substituted by Nb atoms as efficient acceptors^16–18^, we build the MoS_2_ ML with n-p codoping by first substituting one of the Mo atoms in the 3 × 3 supercell with an Nb atom, marked as Nb_1_ in Fig. 1(a). Another Nb atom (Nb_2_) is then considered to adsorb at the surface of the MoS_2_ ML. Totally four typical adsorption sites are explored for the Nb_2_ atom: *H* (Hollow), *M* (Mo-top), *N* (Nb_1_-top), and *S* (S-top) (Fig. 1(a))^43^. Some other adsorption sites, far away from the Nb_1_ atom, are also considered, which are, however, found being unfavorable in energy, compared with the above corresponding sites. The calculations of the electronic structures are performed by using first-principles methods^44^. The exchange-correlation interaction is described with the metaGGA with SCAN form^45^. The energy cutoff is set as 450 eV. Monkhorst-Pack k-point meshes with 5 × 5 × 1 are adopted for structural relaxation and electronic structure calculations. The experimental lattice constant of 3.16 Å^46^ is used and the vacuum space along the z direction is set about 20 Å. The test calculations show that the Nb dopants can expand slightly the lattice structure. Since the energy bands are found insensitive to the lattice constant, the experimental lattice is employed in the subsequent calculations. The convergences of the total energy and Hellmann-Feynman forces are set to be 10^−6^ eV and 0.01 eV/Å, respectively.
